# Evaluation of CO_2_ emissions and energy use with different container terminal layouts

**DOI:** 10.1038/s41598-021-84958-4

**Published:** 2021-03-09

**Authors:** Muhammad Arif Budiyanto, Muhammad Hanzalah Huzaifi, Simon Juanda Sirait, Putu Hangga Nan Prayoga

**Affiliations:** 1 Department of Mechanical Engineering, Universitas Indonesia, Kampus Baru UI Depok, Depok, West Java 16424 Indonesia; 2 Monohakobi Technology Institute, Tokyo, 100-0005 Japan

**Keywords:** Environmental impact, Carbon capture and storage, Mechanical engineering

## Abstract

Sustainable development of container terminals is based on energy efficiency and reduction in CO_2_ emissions. This study estimated the energy consumption and CO_2_ emissions in container terminals according to their layouts. Energy consumption was calculated based on utility data as well as fuel and electricity consumptions for each container-handling equipment in the container terminal. CO_2_ emissions were estimated using movement modality based on the number of movements of and distance travelled by each container-handling equipment. A case study involving two types of container terminal layouts i.e. parallel and perpendicular layouts, was conducted. The contributions of each container-handling equipment to the energy consumption and CO_2_ emissions were estimated and evaluated using statistical analysis. The results of the case study indicated that on the CO_2_ emissions in parallel and perpendicular layouts were relatively similar (within the range of 16–19 kg/TEUs). These results indicate that both parallel and perpendicular layouts are suitable for future ports based on sustainable development. The results can also be used for future planning of operating patterns and layout selection in container terminals.

## Introduction

As a logistics hub between land and sea transportation, container terminals perform an absolutely critical function in the seaborne trade. As a result of the continuous global competition in this field, port operators are currently focusing on sustainable development of container terminals^1,2^. Two of the extremely acute problems requiring urgent solutions are environmental pollution and global warming^3,4^. Thus, reducing CO_2_ emissions and energy consumption at the container terminal is essential to mitigate these environmental impacts^5^. Accordingly, several countries have set targets for reducing CO_2_ emissions per unit throughput at the container terminal^6–9^. Currently, the construction of modern container terminals adopts the green port perspective. A green port refers to a port having a sustainable environment, a fair use of resources, low energy usage and low emissions^10,11^.

Several container terminals have carried out plans of action for emission mitigation and energy efficiency. In order to these plans, several container ports have carried out technological developments such as electrification of container handling equipment^12^, power saving of reefer containers^13^, and the use of alternative fuels also renewable energies^14^. Ports around the world used different methods to calculate the carbon emissions. For example, Port Phillip, Australia and the US Port of Long Beach used the air emission inventory method to calculate emissions^15,16^. The ports of Taipei also calculated their emissions using an activity-based emission model^17^. Similar methods of clarifying entire port-related works by all modes of transport were used in the port of Busan^18^. Other typical methods have also been used in the Port of Los Angeles, USA, which accounts for carbon emissions from all types of active vessels and all equipment used in the port. These data were then analysed to determine the potential emission reductions^19,20^. A research review of the development of maritime logistics in green ports has been carried out using extensive bibliometric and network analysis tools^21^. In addition, a bottom-up study was carried out in Nanjing Longtan Container Terminals, involving the conditions, specifications and burden factor of the equipment as well as the modification of the emission factor^22^.

Moreover, a direct method has been proposed for estimating the carbon emissions based on the distance travelled by the vessels while accounting for all components in the port^23,24^. Other researchers compared rubber-tired gantries (RTGs) to electrical RTGs coming out of the point of view of CO_2_ reduction and energy conservation^25^. Other methods for measuring emissions are based on estimating the vehicle pollution factors according to geometric and traffic conditions, taking into account the basic activities of the vehicle along with the duration of the journey^26–28^. On the other hand, the distribution of emissions from the production activities of ports can be estimated using emission burden inventories and the record of maritime transport activities in ports. The methodology for estimating emissions from light freight vehicles and passenger were conducted by checking the fuel use, throttle position, engine speed, oil temperature and engine coolant^29^. Emission reduction was estimated based on the energy consumption of RTGs, automatic stacking cranes (ASCs) and yard trucks^30^. Using a renewable power source for container-handling equipment achieved significant emission reductions^31^. Approximately 55% of the total emissions in a port are from ships. Thus, it is also necessary to measure emissions from berthing as the ship's auxiliary engine continues to function during loading and unloading^32^. The energy factor required by an additional engine is 40% during berthing^33^.

Very few studies have addressed the effects of terminal layouts on the energy consumption and CO_2_ emissions. Research on container layout design generally investigates resource allocation, optimisation of block length or width and selection of operating technologies. The technologies utilized in these investigations involve simulation experiments and derivation of mathematical formulas. Terminal layouts were studied in one study, taking into account the effect of heap extent and total of layers in a section using a straightforward rule to assess the anticipated amount of repetitions concerning random sampling in the stacking field with regression analysis^34^. An arbitrary design of container fields that considers a layout with transfer paths was introduced in other studies using integer linear programmes. Another model involving an integrated queue network method was applied to investigate 1008 parallel arrangement configurations, resulting in container terminals with parallel arrays that exhibited up to 12% enhanced performance regarding container throughput time compared to terminals with upright heap arrangement^35^. Simulation was also used by several researchers to overcome the high uncertainties in complex terminal systems^36^. Simulation studies evaluated the impacts of yard block layout parameters, such as the height, width and length of the block, and quadruple crane-attached-stand-crane structures, on terminal performances and the yard^37^. As calculated in regard to the work regular level of the cranes, the general long-term efficiency of the port container terminal is affected by the breadth of the depository block in the terminal container yard^38^. An optimisation framework based on simulations was also suggested to acquire an economical and dependable layout solutions that take into account the distribution method of yard equipment and physical arrangement in container terminals^39^. Based on this research, terminal layouts were divided into two categories, namely parallel layouts, which include the majority of layouts currently available, and perpendicular layouts, which are layouts used in automated container terminals that are still in the development phase^40^.

However, most researchers have discussed the optimisation of container block usage and the use of available resources. Very few studies have discussed energy consumption and the resulting CO_2_ emissions. Thus, the aim of this study is to estimate the energy consumption and CO_2_ emissions at different container terminal layouts. Energy consumption was evaluated in a year operation and CO_2_ emissions were estimated based on its energy consumptions and movement-per-modal methods. As a case study, two major container terminals in Indonesia were evaluated. The contribution of this paper is twofold, first is to verify the estimation model of CO_2_ emissions in container terminals, where the movement-per-modal method was verified with emission based on energy consumption. The second is more interesting is to provide an overview of the feature of parallel and perpendicular layouts of the container terminal in terms of CO_2_ emissions.

## Research and methodology

### Estimation of energy consumption

Energy consumption was estimated based on the fuel and power consumptions of each container-management machinery working in the terminal during a certain extent of time. The container-management equipment at the terminal are grouped into three main categories: container cranes, head trucks for container transportation and equipment for container handling in the yard such as automatic stacked crane, straddle carrier, reach stacker and side loader. Energy used in container terminals are obtained from the electricity and fuels, mainly diesel. Container cranes are the only equipment that uses electricity. Here, energy consumption data was obtained from historical records of the fuel and electricity consumptions at the destination terminal. The data collection method involved the observation of operation performance of the handling equipment in the container terminal over a year. Following are the types of information collected:

1. Equipment specifications and utility data. These data included the number of container-handling equipment at container terminals, specifications of each of the container-handling equipment (i.e. engine manufacturer data, equipment capacity, year of manufacture and fuel oil consumption) utility data (i.e. the operational time (in hours) of each container-handling equipment and equipment utility in 1 year). The equipment utility was calculated built upon the number of hours the container-management machinery was used in 1 year minus the required time for maintenance and breakdown operation.
2. Fuel and power consumptions. These data include the quantities consumed of diesel fuel (litre) and electricity (kWh). The data were collected based on the container port records for each container-handling equipment. In case of no recorded data, the fuel consumption was calculated using a consumption factor for each container-handling equipment.

After data collection and storage, energy consumption at the container terminal was estimated using Eq. (1), where the sum of all energy used in container cranes, container-handling equipment and head trucks. Equation (2) was used to estimate the energy used during container handling in the terminal area, which is the sum of all energy used by automated stacker cranes, straddle carriers, reach stackers and side loaders. The energy consumption for each equipment was calculated using Eq. (3) based on the utility time of the equipment multiplied by fuel consumption.

1 $$E_{port} = E_{cc} + E_{ch} + E_{ht}$$

2 $$E_{ch} = E_{asc} + E_{rtg} + E_{sc} + E_{rs} + E_{sl}$$

3 $$ET = UT x FC$$

wherein E_port_ = Energy consumption the container terminal, E_cc_ = Energy used by the container cranes, E_ch_ = Energy used by container-handling equipment, E_tt_ = Energy used by terminal trucks, E_asc_ = Energy used by automated stacking cranes, E_sc_ = Energy used by straddle carriers, E_rs_ = Energy used by reach stackers, E_sl_ = Energy used by side loaders, ET = Energy used by each container-handling equipment, UT = Utility of equipment in a 1-year operation, FC = Fuel consumption by container-handling equipment.

### Method of estimating CO_2_ emissions

The method of estimating CO_2_ emissions at container ports is carried out in two ways, namely calculating based on energy consumption and based on modality movement. Calculation of CO_2_ emissions based on energy consumption is done by multiplying the energy consumption data by the emission factor of each type of equipment, this method requires recorded data of fuel and power consumption from the container terminals. The second way is to estimate CO_2_ emissions based on modality movement, the emissions caused by transhipment in a container terminal can be mapped using the emissions-per-modal method. Since the energy used in transhipment processes results in CO_2_ emissions, the factors affecting the transhipment process. This includes the energy consumed by all the machinery utilised in every sub-method, energy use patterns of differing container-handling machinery, equipment distribution and the average distance in each sub-process. This method was introduced by Van Duin (2011) aims to minimise the use of recorded data from the container terminals to enable the estimation of macro emissions^24^. However, the input variables to this model should be close to the actual conditions, which include:

1. *Total throughput containers in 1 year*
  In this performance model, the full container utility should be recorded all can be represented by the containers dealt with.
2. *Transportation modality*
  The movement modality must be monitored with respect to the distribution of the total throughput containers based on various modalities. The process of handling containers and their routes depends on the type of modality used.
3. *Transhipment process*
  Transhipment processes within terminals vary depending on the types of modalities in the terminals. The process of moving containers, types of equipment used and modalities are part of the transhipment process.
4. *Terminal layout*
  The energy consumption by machinery relies on the ranges to and from the sub-methods, too. Thus, the container terminal location determines the above-mentioned ranges. Every terminal carries its particular layout, in which the ranges depend on the differing areas of equipment in the terminal. Therefore, energy use was measured utilising the ordinary range based on the kinds of machinery per procedure as well as the distances to and from the stacking area, jetty, gate and other points, which were determined according to the satellite photo. The Manhattan-distance metric system was used in the calculations.

This estimation system utilises the results of the bottom-up calculations of the work activities performed in the port and fuel consumption and does not consider them as input variables. The container movement and rides are the activities considered in these calculations. Container movement is the movement by a truck as the transportation method so that an additional variable is needed, which is the distance calculated using the Manhattan-distance metric system. A ride on the other hand is the movement by means of a crane, stacking crane, RTG or another container-handling equipment. After considering the modality movement, the total CO_2_ emissions in a container terminal was predicted based on the amount of emissions produced by a combination of various equipment and the contribution of each equipment to the movement sub-processes (Eqs. 4, 5 and 6). Table 1 shows the patterns of energy use of differing container-handling machinery calculated using these equations.

4 $$W_{x} = \mathop \sum \limits_{i = 1}^{5} \mathop \sum \limits_{J = 1}^{1} \left( {\left( {{\text{V}}_{i,j} \times f_{D} } \right) + \left( {P_{i,j} \times f_{E} } \right)} \right)$$

**Table 1:** Energy consumption patterns of each container-handling equipments.

Power source	Kinds of container-handling machinery	Usage variable*
Diesel	Rubber-tired gantry cranes (RTGs)	1.32 L/move
	Straddle carrier (SC)	3.50 L/km; 0.80 L/move
	Terminal head trucks (TT)	3.23 L/km
	Automated terminal tractor (ATT)	1.67 L/km
	Reach stacker/top (RS)	5.00 L/km
Electricity	Quay crane (Qc)	6.00 kWh/move; 2.77 L/move
	Ship to shore (STS)	6.70 kWh/move
	Automated Stacking crane (ASC)	5.00 kWh/move

*This variable was estimated according to previous research^24,41,42^.

Combined with:

5 $$V_{ij} = n_{ij} *\left( {C_{ij} + c_{ij} + X_{ij} } \right) \forall^{i,j} \in T$$

6 $$P_{ij} = n_{ij} *\left( {p_{ij} } \right) \forall^{i,j} \in T$$

in which W_x_ = The amount of CO_2_ emissions weight generated at terminal x, V_i j_ = The annual diesel use in litres by equipment *i* in modality *j*, f_D_ = The factor of emission in kg of CO_2_ emissions per a litre of diesel (= 2,65), P_i j_ = The annual power use in kWh by equipment *i* in modality *j*, f_E_ = The factor of emission in kg of CO_2_ emission per kWh (= 0.832), n_(i,j)_ = The amount of trips of equipment *i* in modality *j*, C_(i,j)_ = The steady consumption (e.g. hoisting works) per trip in litres, c_(i,j)_ = The variable consumption per km in litres, $X\overline{{_{ij} }}$ = The range crossed by equipment *i* in modality *j*, p_ij_ = The steady consumption per trip in KWh, *i* = Type of equipment, in this case, the study consist of 5 types of equipment depends on its terminals, *j* = Type of modality, in this case, the study consists of 1 type of modality which is inter-terminal transport, *T* = Container terminal.

In this study, the emission factor of diesel fuel was obtained from the guidelines of the nationwide greenhouse gas inventory issued by IPCC 2006 or the Intergovernmental Panel on Climate Change^43^. The electricity grid emission factor was obtained from the national electricity company servicing each designated port. Table 2 shows the emission factor of each energy source.

**Table 2:** The emission factors used for the container-handling equipment and auxiliary engines of the ships.

Energy source	CO_2_ emission factors	Reference
Industrial diesel oil	2.67 kg/L	IPCC 2006^43^
On grid electricity	0.84 kg/kWh	National electric company^44^

### Characteristic of container terminal layouts: a case study from two major container terminals in Indonesia

**Figure 1:**
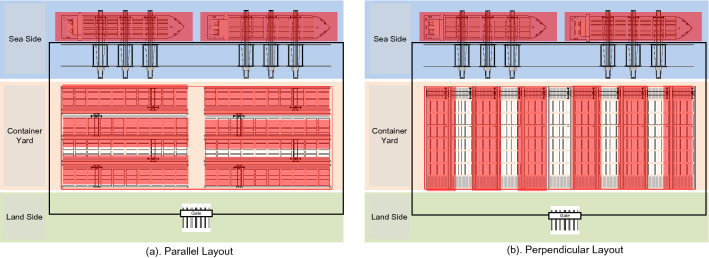
Typical layouts of container terminals.

As a case study, two major ports in Indonesia with different types of terminal layouts (i.e. parallel and perpendicular) were investigated. In the parallel layout, blocks are arranged side-by-side to the dock and transfer points situated next to a gulf per section. This design is widely used in Asia. In the perpendicular layout, blocks are arranged perpendicularly to the dock as well as the transfer points situated at the two edges of each section. This layout is widely used in Europe and most modern terminals. Figure 1 shows a container terminal upon side-to-side and upright heap designs. Here, the two studied terminals were called container terminal A and container terminal B. The two terminals are Indonesian state-owned companies. Terminal A has a parallel layout and terminal B has a perpendicular layout. Table 3 shows the characteristics and number of container-handling equipment of these terminals. Based on these characteristics, the terminals were categorised according to the order of annual throughput. Thus, terminal A and B were considered a large and a medium container terminals, respectively. As geographically, the features of terminals A and B have different characteristics, Terminal A with a parallel layout is located in the bay with face to the open sea, in general, the location of the berth is side by side with the container yard. While Terminal B with the perpendicular layout is into the estuary or canal, in general, the location of the berth protrudes into the shipping channel, in its development, this feature is a typical deep seaport^45,46^.

**Table 3:** Container-management facilities in the container terminals.

Terminal characteristics	Container terminal A	Container terminal B
Layout types	Parallel layout	Perpendicular layout
Throughput	1,596,140 TEUS/year	637,733 TEUS/year
**Berth data**		
Length (m)	1640	950
Width (m)	26.5–34.9	30–50
Depth (m)	12–16	13–14
**Container yard**		
Area	455,000 m^2^	252,000 m^2^
**Number of equipment**		
Container crane	16	10
Rubber-tired gantry crane	63	–
Automatic stacking crane	–	20
Head truck	103	50
Reach stacker	4	4
Side loader	6	3
Straddle carrier	–	5
Specific characteristics	The terminal uses fossil fuel as an energy source for the rubber-tired gantry crane	The terminal uses electric power as an energy source for the automatic stacking crane
Satellite imagery		
	Retrieved from Map Yahoo Japan on 23rd October 2020^47^	Retrieved from Map Yahoo Japan on 23rd October 2020^48^

## Result and discussion

### Result of energy consumption at container terminals

**Figure 2:**
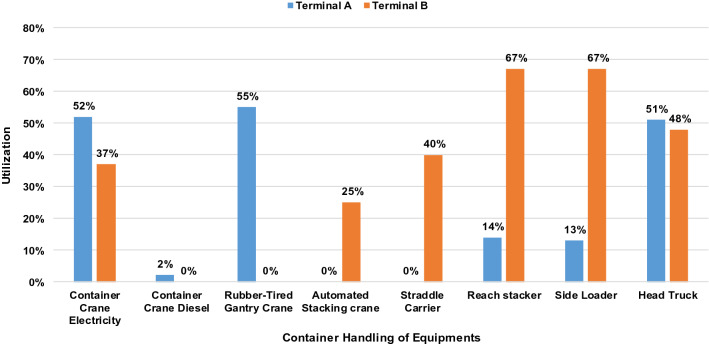
Utility of container-handling equipment observed in the container terminals.

In this section, the utility data obtained from the container terminals (Fig. 2) are discussed in relation to the results of the estimated energy consumption. The utility graph in Fig. 2 shows that the two terminals exhibited different characteristics in terms of the number of instruments and port operations. Terminal A is classified as a parallel layout with an RTG crane as a loading and unloading equipment in the stacking yard, while Terminal B is a terminal with a vertical layout that uses an ASC as a container-handling equipment. Similar to the majority of container terminals, container cranes, head trucks and RTGs are the most utilised in terminal A, which is consistent with previous studies. However, in terminal B, reach stackers, side loaders and straddle carriers are the most utilised, which is consistent with other studies. The interesting finding of terminal B is that reach stacker and side loader are the most utilised equipment. This can be attributed to the buffer area used by the container stacking system in terminal B, where the stacking location can be easily adjusted.

**Figure 3:**
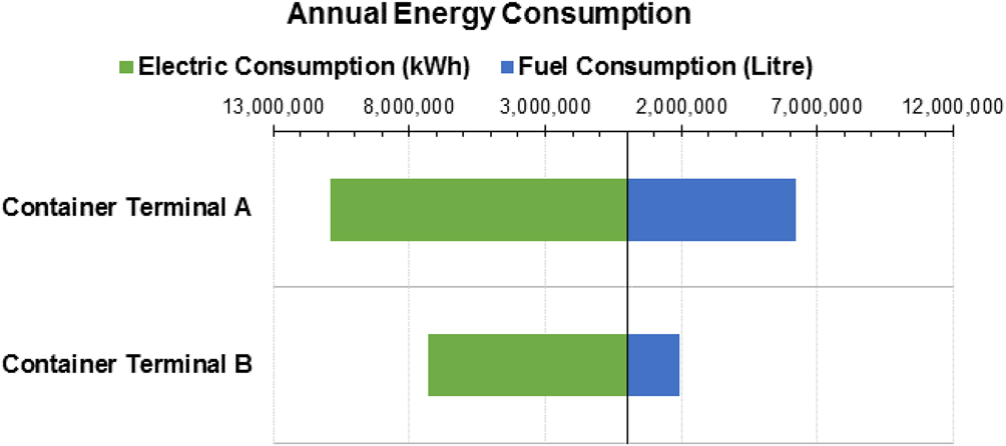
Annual energy use of container terminals.

Annual energy consumption data consisting (i.e. electricity and fuel consumption) are obtained from the two terminals. Figure 3 shows the annual energy consumption of the studied container terminals. The two terminals exhibited different consumption patterns because their operational capacity is different as terminal A has a higher throughput capacity than terminal B.

**Figure 4:**
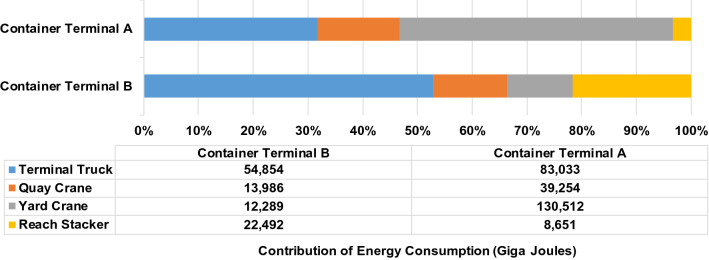
Contribution of container-handling equipment to the energy consumption at the container terminal.

Figure 4 displays the contribution of each container-handling equipment to the energy consumption in giga joule. The figure indicates that, in container terminal A, the contribution of the equipment to the energy consumption was in this order: the RTG cranes, truck terminals and quay cranes. In container terminal B, the contribution of the equipment to energy consumption was in the following order: truck terminals, reach stackers and quay cranes. Truck terminals contribute the most to the energy consumption of Terminal B because this terminal has a relatively far berth and yard location connected by the harbour trestle.

### CO_2_ emission based on movement modality

**Figure 5:**
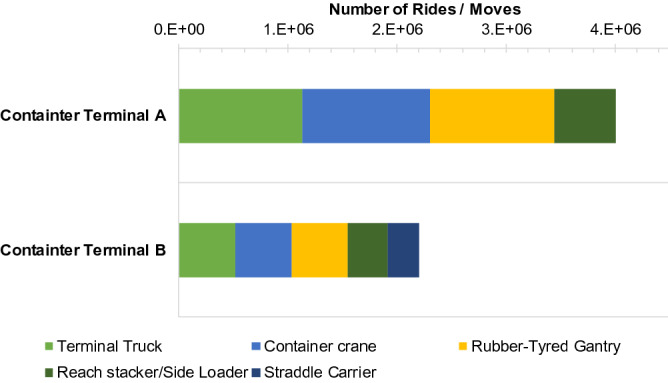
Number of rides on container-handling equipment.

The number of container transfers by each device at each container terminal is an important variable in these calculations. Figure 5 shows the inventory data of each container terminal regarding the number of rides/moves carried out by each device. In the truck terminal, the data required include the number of movements in the process of carrying a container (under ideal conditions, this number will be the same as the number of boxes per year at the container terminal). The number of movements under ideal conditions is the number of boxes added to the number of hatch covers that must be opened and closed during the export or import process in ship to shore (STS) cranes and container cranes. In RTGs and ASCs, the number of movements is similar to the total containers added to the total shifting that occurs during the export or import process. This is the same process performed by straddle carriers and reach stackers.

**Figure 6:**
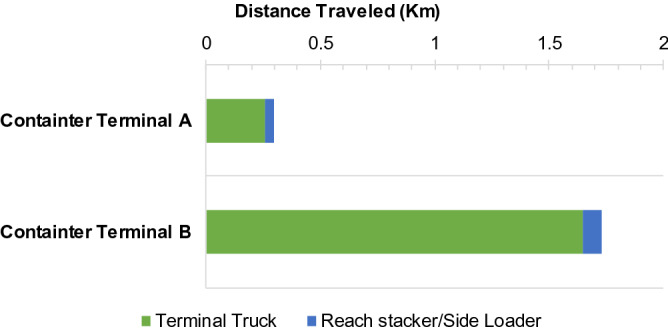
The distance travelled from the ship's point of loading and unloading by the container crane to the stacking yard.

The distance travelled by an instrument to move containers is also an important variable in calculating port operational emissions. Figure 6 shows the distance travelled by each container-handling equipment at the container terminals. The distance travelled by terminal trucks was estimated from the loading/unloading point of a ship by container cranes to the stacking yard at the point where the container is handled by RTG. For reach stacker/side loader the distance was considered that between the points at the corners of the container stacking yard.

**Figure 7:**
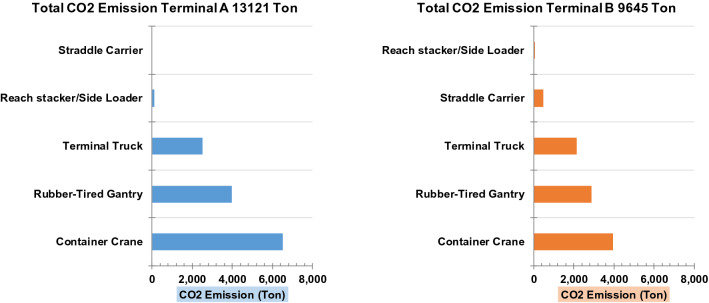
CO_2_ emissions generated by container-handling equipment calculated based on the movement modality.

Based on these results, the contribution of each container-handling equipment to the CO_2_ emissions can be calculated. Figure 7 shows the contribution the contribution of each container-handling equipment to the CO_2_ emissions. The CO_2_ emissions calculated using movement modality over 1 year of operations in terminals A and B were found to be 13,121 and 9645 tonnes, respectively. The total CO_2_ emissions in a container terminal depend on the total of containers managed by each container-handling equipment. In container terminal A, GTR cranes, quay crane and truck terminal exhibited the largest emission contributions, which is consistent with the energy consumption of each equipment. In container terminal B, truck terminals, container cranes and ASCs exhibited the largest emission contributions, which is also consistent with the energy consumption by each equipment. Thus, the contribution to CO_2_ emissions at container terminal depends on the number of movements of the container-management machinery. The number of movements is influenced by several factors such as the number of containers, container layout, operation pattern and operation distance.

**Figure 8:**
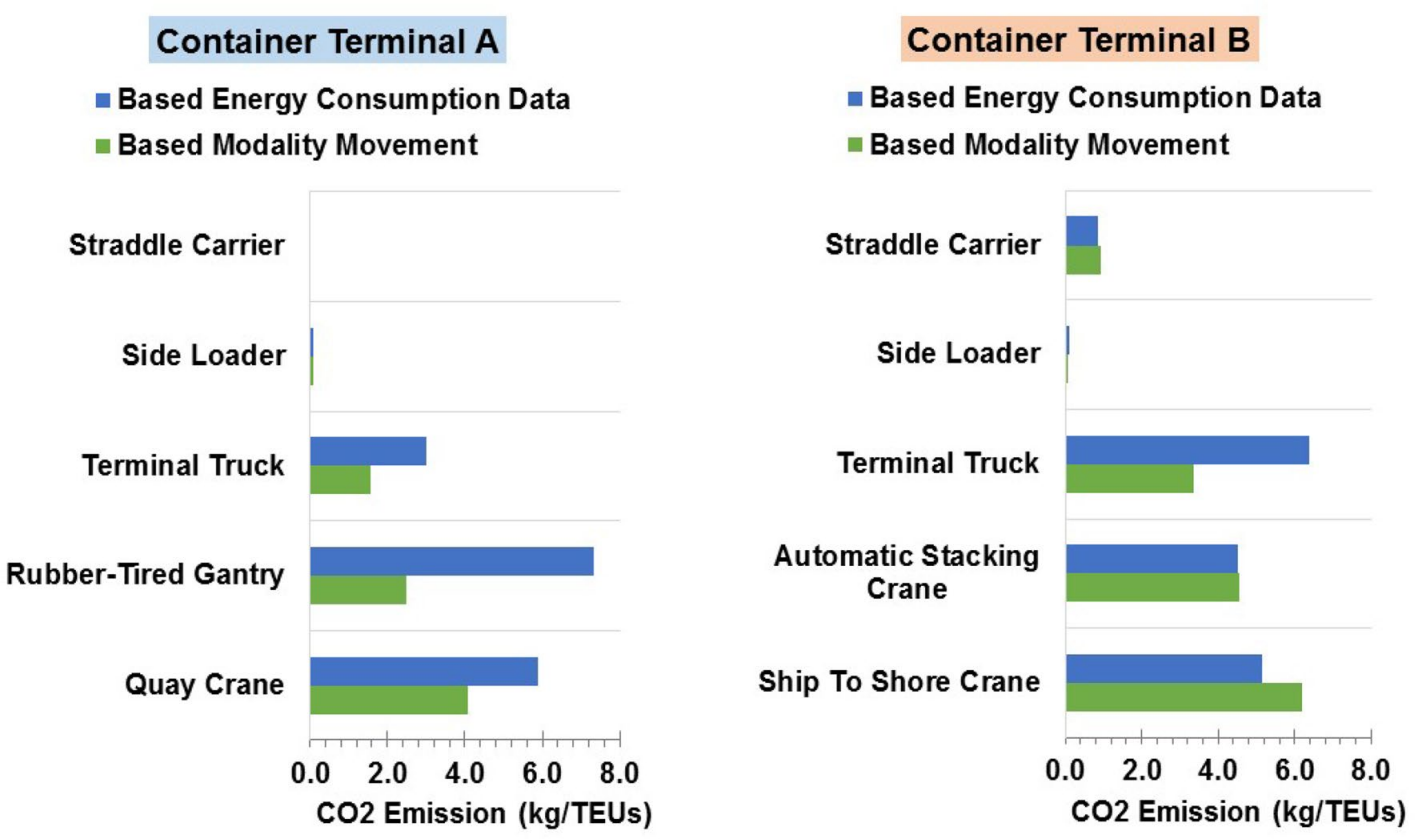
Comparison between CO_2_ emissions arising out of container-handling equipment built upon energy use data and those based on movement modality.

Moreover, the CO_2_ emissions estimated based on the movement modality were compared to those predicted built upon the conversion of power consumption using the emission factor of each energy type (viz. the power use increased by the emission factor). Here, the emission factors were assumed to be 2,65 kg/L and 0.832 kg/kWh for diesel oil and electricity, each. Figure 8 displays the outcomes of the estimated CO_2_ emissions based on movement modality compared to those based on the conversion of energy consumption by emission factors. These results show that the results of CO_2_ emissions calculated by movement modality agreed well with the result of those based on the conversion of energy consumption. The closest results were obtained in terminal B, where the close results of the emissions from STS cranes and ASCs were obtained in both cases.

### CO_2_ emissions per twenty-foot equivalent units (TUEs) at the container terminal

**Figure 9:**
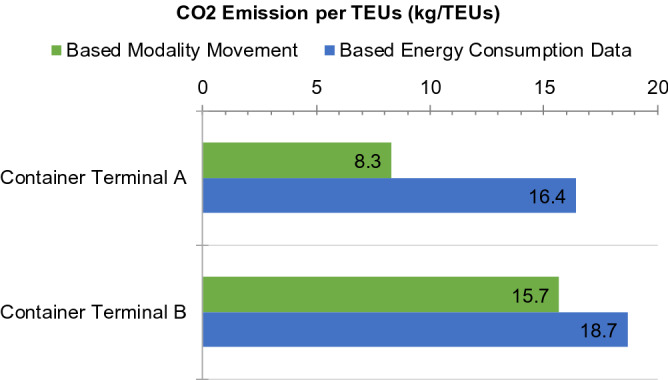
Comparison between the total CO_2_ emissions per TEUs at the studied container terminals.

In this section, the CO_2_ emissions per TEUs were discussed. This value provides an overview of the emissions generated by each container movement at the container terminal. Figure 9 shows the CO_2_ emission per TEUs at the two terminals. The results show that the CO_2_ emissions based on energy consumption in Terminals A and B were 16.4 and 18.7 kg/TEUs, respectively. Although terminal B has a slightly higher value, the values are still close because the distance between the vessel berth and the area of the stacking yard is larger in terminal B. Based on these results, the CO_2_ emission contribution per container is quite equivalent in the two types of terminal layouts i.e. parallel and perpendicular, even though Terminal B has a perpendicular layout, which is claimed to be the more modern layout. Thus, this indicates that the parallel layout is a better option, which is important for future planning of container terminals.

**Figure 10:**
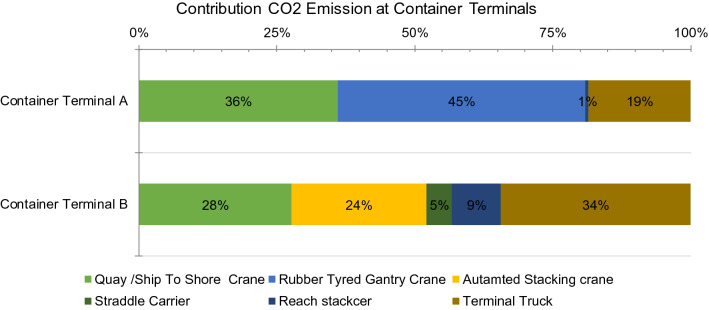
Contribution of each container-handling equipment to the CO_2_ emissions at the studied container terminals.

CO_2_ emissions at the container terminal are the result of using container-handling equipment. Each container-handling equipment contribute a part of these emissions depending on the characteristics of the terminal. Figure 10 shows the contribution of container-handling equipment to the CO_2_ emissions at each container terminal. These results show that each container terminal has different characteristics based on the layout, which affects the operational process at the terminal. In container terminal A, RTG cranes exhibited the largest contribution (approximately 45%) to the total CO_2_ emissions because this terminal has a large container throughput; thus, the container traffic volume in the stacking field is also high, which indicates that this equipment experiences several container re-handlings. However, in container terminal B, the biggest contribution was that from the terminal truck (approximately 34%). However, this problem is unique to this particular terminal because the distance between the ship berth and stacking yard is quite large. This needs to be considered in future preparation of container ports.

**Figure 11:**
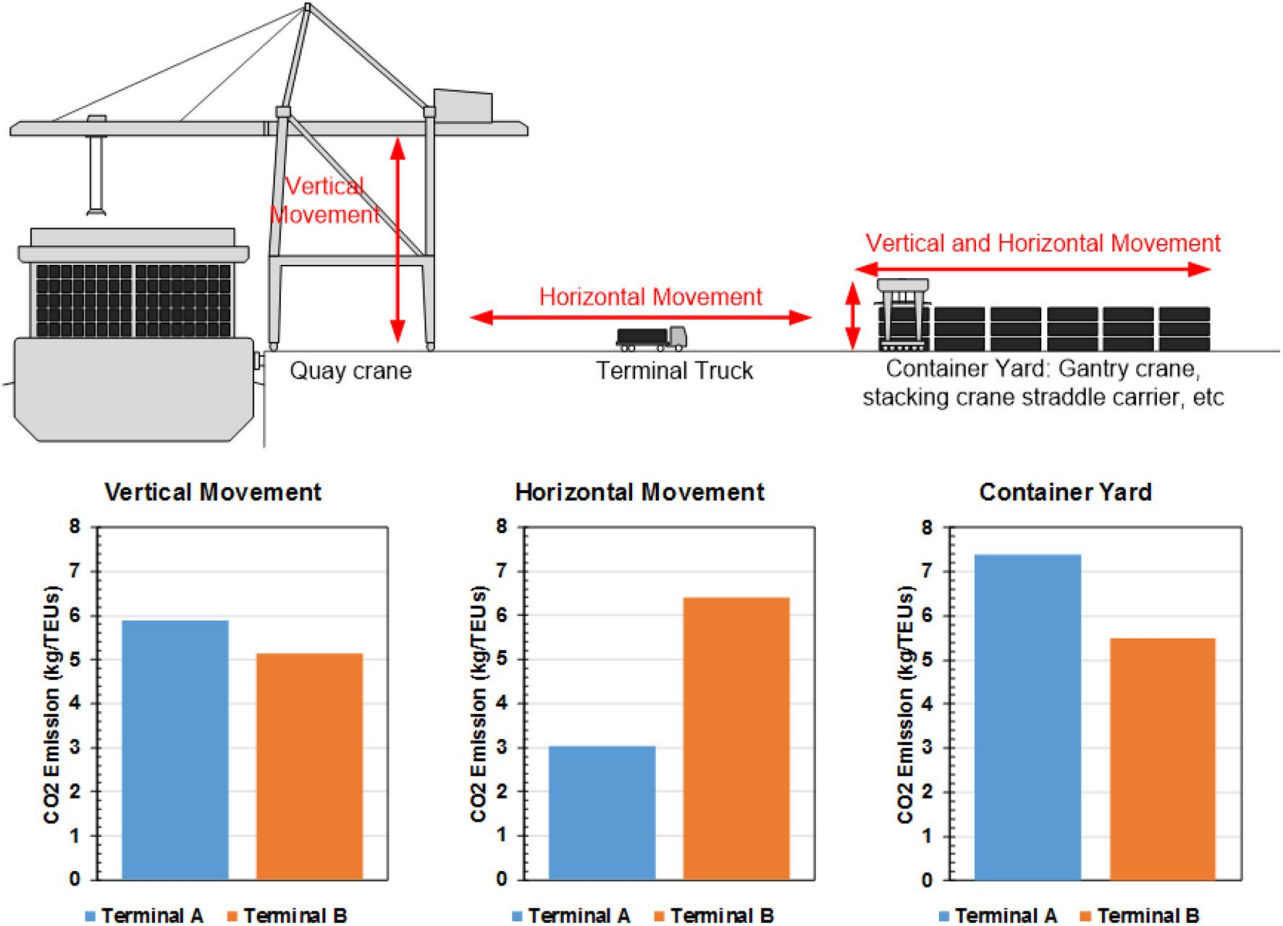
Contribution of each container-handling equipment to the CO_2_ emissions at the studied container terminals.

This section discusses the CO_2_ emissions in terms of port operation area. There are three main operations in container terminals: vertical movement, horizontal movement and yard operation. An overview of the contributions of these areas to the CO_2_ emissions is shown in Fig. 11. These results show that the dominant contribution to emissions occurred in the yard operation area followed by that by the vertical movement. The contribution of horizontal movement to the emissions in terminal B was not considered since the range between the yard and dock is quite large. CO_2_ emissions from every container-handling equipment, even in the operation area, can form a baseline for CO_2_ emissions, which can be used to achieve the purpose of cutting down greenhouse gas emissions. As announced by IMO or International Maritime Organization agreement, the Marine Environment Protection Committee reported that the shipping industries agreed on a target of reducing the total CO_2_ emissions by 50% by 2050 and beginning the emission cuts as soon as possible^49^, while pursuing efforts to completely phase out carbon emissions.

### Uncertainty analysis of CO_2_ emissions

This section discusses the results of the uncertainty analysis of the CO_2_ emission calculations at the container ports based on the energy consumption model and modality movement model. Uncertainty analysis is carried out in two stages using statistical parameters. The first stage is the calculation of the standard uncertainty of each type of container-handling equipment in the terminal, and the second stage is measuring the robustness of the emission calculation model based on the energy consumption and modality movements. The standard uncertainty (u) is calculated based on the standard deviation (σ) of the energy consumption for the container-handling equipment and the number of independent observations (n)^50,51^. The number of observations is the energy consumption recapitulated every month in 1 year, so there are 12 observations of recap data for each type of container-handling equipment. The results of the standard uncertainty analysis from the container-handling equipment are used to ensure that the energy consumption data have a significant effect on the total emissions. The robustness of the emission calculation model is indicated by the R-squared value after the emission calculation results are regressed on the energy consumption and modality movement. When the regression is significant, the R-squared value indicates how much the emission calculation is affected by the energy consumption and the modality of movement.

Tables 4 and 5 show the standard uncertainty of each type of container-handling equipment in Terminals A and B, respectively. The results of the standard uncertainty for the container-handling equipment in both Terminals A and B showed low uncertainties; some equipment had relative uncertainties below 5%. In Terminal A, there were three types of equipment that had low relative uncertainties and high R-squared values (above 0.7), namely, the quay crane, rubber-tired gantry crane, and terminal truck. These results indicate that these three types of equipment had a major effect on the total emissions at Terminal A. In addition, they also had high energy consumption.

**Table 4:** Uncertainty results for terminal A.

Terminal A	Mean	SD	Uncertainty	%Relative uncertainty	R square
Quay crane	782.8	80.4	23.2	3.0	0.74
Rubber-tired gantry crane	933.6	81.0	23.4	2.5	0.80
Terminal truck	401.8	40.5	11.7	2.9	0.93
Reach stacker	12.5	3.4	1.0	7.9	0.57
Side loader	12.2	4.4	1.3	10.3	0.35

**Table 5:** Uncertainty results for terminal B.

Terminal B	Mean	SD	Uncertainty	%Relative uncertainty	R square
Ship to shore crane	273.2	20.4	5.9	2	0.75
Automatic stacking crane	240.1	21.6	6.2	3	0.91
Terminal truck	339.7	28.9	8.3	2	0.80
Straddle carrier	45.8	7.5	2.2	5	0.64
Side loader	5.7	4.3	1.2	22	0.05

The results of the standard uncertainty also showed similar results in Terminal B. In that terminal, there were also three types of equipment that had low relative uncertainties and high R-squared values (above 0.7), namely, the ship-to-shore crane, automatic stacking crane, and terminal truck. Based on the similar results of the uncertainty standard in both of the terminals, it can be concluded that there were three types of equipment in each terminal that had a major effect on the total emissions. These three types of equipment represent the three movements that are in the container terminal, namely, vertical movement (the quay and ship-to-shore crane), horizontal movement (terminal truck), and a combination of vertical and horizontal movement (rubber-tired gantry crane and automatic stacking crane).

**Figure 12:**
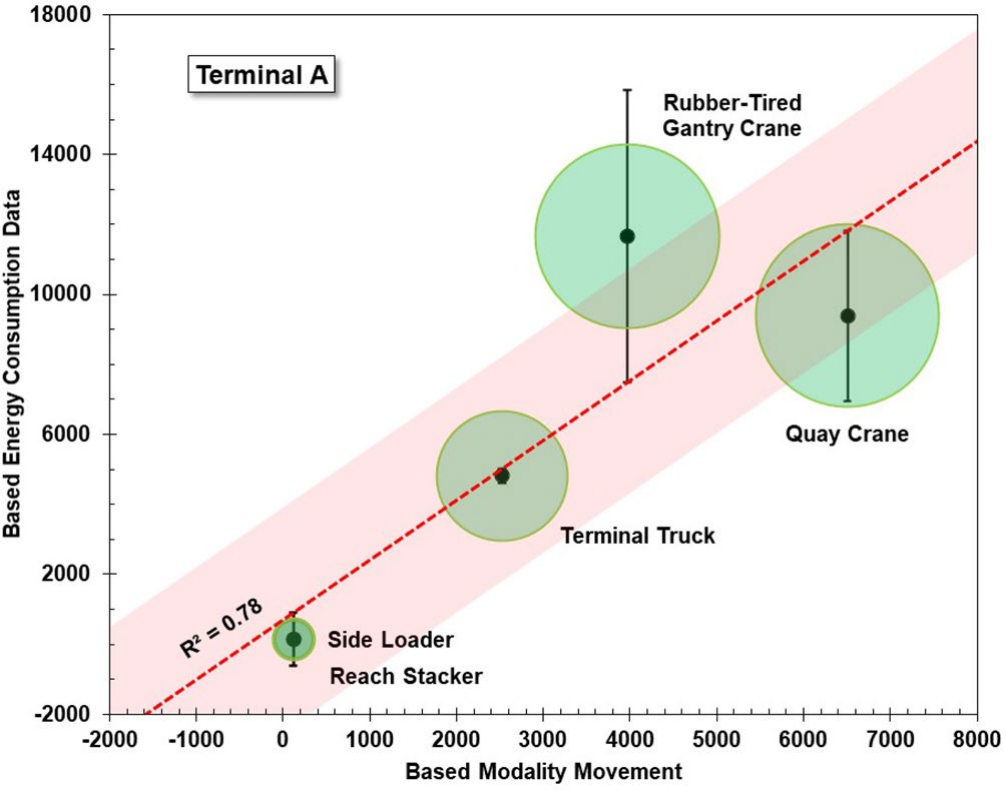
Robustness of the CO_2_ emission calculation model for terminal A.

**Figure 13:**
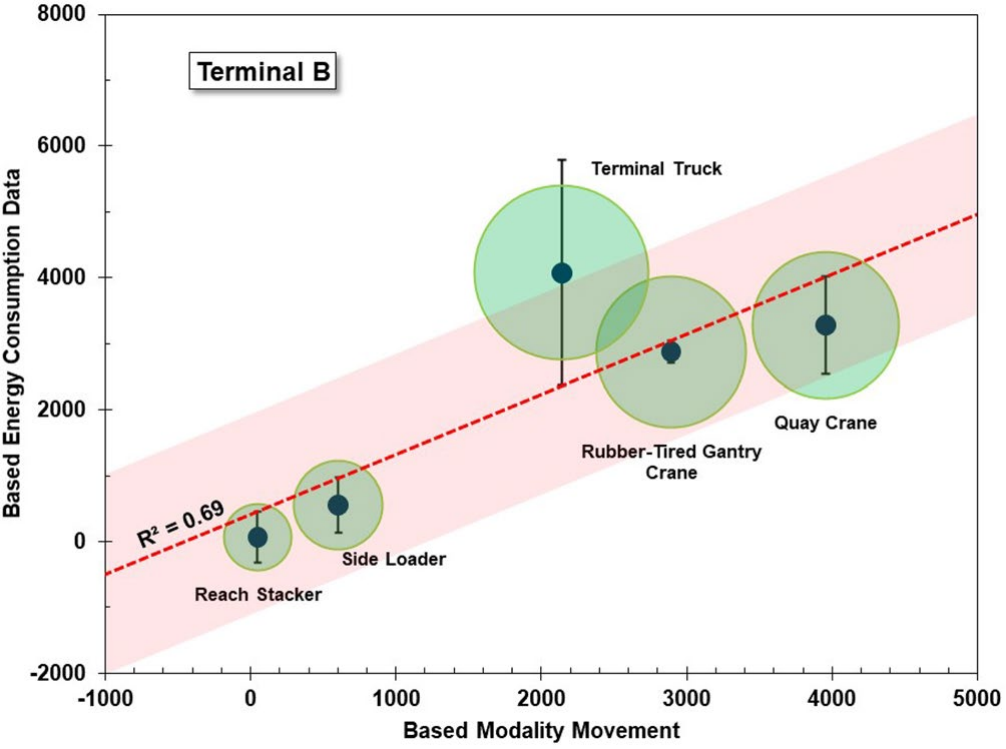
Robustness of the CO_2_ emission calculation model for terminal B.

Figures 12 and 13 show the robustness of the CO_2_ emission calculation models based on the energy consumption and modality of movement for Terminals A and B, respectively. The robustness of the model is measured using a regression equation with a 95% confidence level. The results show that the R-squared values for Terminals A and B were 0.78 and 0.69, respectively. This value is considered strong enough to indicate a level of robustness in the calculation of CO_2_ emissions, where an R-squared value above 0.6 indicates a strong correlation between the two variables^52^. Based on the correlation results, interesting findings were obtained for both Terminals A and B. In Terminal A, it was found that the emission results for the rubber-tired gantry crane showed high error, while in Terminal B, high error was shown for the terminal truck. These results are informative regarding the CO_2_ emission contribution characteristics in the container terminal based on the type of layout. They show that in Terminal A, which is a typical parallel layout, there is a high potential for the uncertainty of the CO_2_ emissions in the vertical and horizontal movement areas. In Terminal B, which is a typical perpendicular layout, the high potential for the uncertainty of CO_2_ emissions is in the horizontal movement area.

### Policy implications: toward sustainable development goals

With more than 80% of global trade by volume carried by maritime transport, In line with the OECD’s industrial production growth rate, the demand for container trade has been increasing over the past decades, driven by an expanding service and retail sector in developed economies. Amid of COVID 19 pandemic experienced globally, the outlook for the container shipping markets remains strong moving into 2021^53^. To cope with this demand, container terminal still plays a key role in sustainable development and prosperity to support this trade volumes.

The key drivers for the container trade demand will consist of structural changes in the industrial and business cycle of emerging economies^54^; reshoring activities motivated by automation and the sustainable development goals (SDGs) that consists of 17 interlinked global goals blueprint to achieve a better and more sustainable future^55^. In order to pursue the target of SDGs, global container terminal players under the International Association of Ports and Harbors along with the International Maritime Organization (IMO) decided to set up a World Ports Sustainability Program, intended to promote the preservation of environments by making continual improvements in operations through design and operation^56^. Moreover, IMO specifically targeting a reduction of CO_2_ emissions reduction by 50% in 2050^49^.

The implication of this research is to set-up a simple yet robust framework to measure energy consumption and CO_2_ emissions at container terminals. Evaluation of energy consumption of the port should start from the selection of layout all the way through investing in energy-efficient port equipment (stationary and mobile material handling equipment, lighting and technology), that will support the operation of the selected layout. Overall, this framework will contribute directly to the 13th Goal, i.e. Affordable and Clean Energy, by improving energy efficiency of port and adapting port infrastructure and port related operations to Climate Change, as well as aiming for the 9th SDG, i.e. Industry, Innovation and Infrastructure by foreseeing the adaptation of port infrastructure to withstand climate change.

In line with the SDGs through the IMO targets, this research suggests an important finding, that the modern layout of terminal found in developed countries doesn’t contribute much to the above-mentioned sustainable goals, more than the selection of the container handling equipment within the terminal and decarbonization of cargo handling. It then very important for the port operator to integrate future layout planning, not only to accommodate market demands and stakeholders’ interests but also strongly consider the projection of CO_2_ emission and energy consumption. The future direction of this research shall address the formulation of standard measurement to incorporate the calculation of emission considering different terminal layout and arrangement of cargo-handling equipment.

## Conclusions

Energy consumption and CO_2_ emissions at container terminals were calculated in different terminal layouts using two existing terminals as a case study. Energy consumption has been calculated based on utility data and fuel consumption as well as electricity consumption of each container-handling equipment. The energy consumption of terminals A and B (with the parallel and perpendicular layouts, respectively) was calculated based an overview of the energy consumption from each equipment. It was found that RTG cranes have the largest contribution to the energy consumption in terminal A (50% of total energy consumption), whereas in terminal B, truck terminals were the largest contributor (53% of total energy consumption). These results show that the terminal layout affects the energy consumption of each container-handling equipment used at the container terminals. In terminal B, the truck terminal exhibited the largest contribution because the ship berth is located far from the container yard, which may not apply to other terminals.

The number of movements and travelling distance form the basis for estimating CO_2_ emissions. These results were compared with those estimated based on the conversion of energy consumption using emission factors. The CO_2_ emissions estimated by the movement modality agreed well with those estimated based on energy consumption data. These results show that the contribution to the CO_2_ emissions in the two studied terminals were associated with the number of containers throughout as the CO_2_ emissions in terminals A and B terminals (16.4 kg/TEUs and 18.7 kg/TEUs, respectively) are relatively equivalent. This indicates that terminal B with a perpendicular layout, which is claimed to be a modern layout, exhibited emission quantities equivalent to those of terminal A, which has a more common parallel layout. These results may shed light on the sustainable development of container terminals because they prove that both layouts are suitable for future development. Furthermore, this study constructs a baseline in furtherance of cutting down CO_2_ emissions at container terminals to achieve the IMO targets that contribute to United Nation’s Sustainable Development Goals.
